# Subsidence of Cerebro-Spinal Meningitis as an Epidemic

**Published:** 1872-07

**Authors:** 


### Editorial.

*Subsidence of Cerebro-Spinal Meningitis as an Epidemic.*

In making the announcement, that "Cerebro-Spinal Meningitis" in its epidemic form, has disappeared from Buffalo and near vicinity, we have little to add; indeed before such a fearful malady our words should be very few. Its appearance and disappearance is shrouded in profound mystery; its progress and termination uninfluenced by any agencies with which we are acquainted, a fearful and dreaded plague, such as has never visited this vicinity before. It is idle to liken it to diseases, with which we are all familiar, for whoever has watched the modes of attack, progress, and termination, see most clearly that it does not resemble in the remotest degree any other disease with which we are acquainted. The attempt to point out any probable or possible causes, has thus far failed, and all that physicians *know* of it, is comprised in what has been learned of the morbid changes which in some cases, and especially in cases of long standing, have been seen. These show that the disease is inflammatory in character, or at least, leads to the deposit of inflammatory products. The remarkable absence of all observable pathological change in some acute cases, cannot be set against, positive proof of its inflammatory character, where it is found. Sporadic cases will, no doubt, be observed, and physicians can never study such cases too attentively; the subject is too full of interest, to escape investigation and inquiry.

